# Observing in space and time the ephemeral nucleation of liquid-to-crystal phase transitions

**DOI:** 10.1038/ncomms9639

**Published:** 2015-10-19

**Authors:** Byung-Kuk Yoo, Oh-Hoon Kwon, Haihua Liu, Jau Tang, Ahmed H. Zewail

**Affiliations:** 1 Physical Biology Center for Ultrafast Science and Technology, Arthur Amos Noyes Laboratory of Chemical Physics, California Institute of Technology, Pasadena, California 91125, USA

## Abstract

The phase transition of crystalline ordering is a general phenomenon, but its evolution in space and time requires microscopic probes for visualization. Here we report direct imaging of the transformation of amorphous titanium dioxide nanofilm, from the liquid state, passing through the nucleation step and finally to the ordered crystal phase. Single-pulse transient diffraction profiles at different times provide the structural transformation and the specific degree of crystallinity (*η*) in the evolution process. It is found that the temporal behaviour of *η* exhibits unique ‘two-step' dynamics, with a robust ‘plateau' that extends over a microsecond; the rate constants vary by two orders of magnitude. Such behaviour reflects the presence of intermediate structure(s) that are the precursor of the ordered crystal state. Theoretically, we extend the well-known Johnson–Mehl–Avrami–Kolmogorov equation, which describes the isothermal process with a stretched-exponential function, but here over the range of times covering the melt-to-crystal transformation.

## Abstract (web summary)

The phenomenon of crystallization is common in nature, but surprisingly the nucleation pathways from liquid to solid are poorly understood due to the lack of effective experimental probes. Yoo *et al*. observe the existence of a nucleation precursor in titanium dioxide using single-pulse electron microscopy.

## Graphical abstract

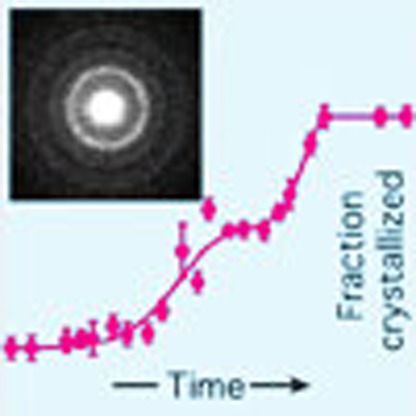

Crystallization, which is an ancient and common phenomena of nature, is an disorder-to-order transformation of a substance^1^. The processes involved in crystallization are complex, depending on the temperature, pressure and particle size, and the timescales are vastly different. For example, it takes minutes to hours to achieve the phase transformation of a bulk material through equilibrium, thermal annealing, whereas the transition using laser non-equilibrium heating can occur on the nanosecond (ns) timescale, 10 orders of magnitude faster than that of equilibrium heating^2^. The nature of the transformation and the timescale of the processes involved has been the subject of many studies. Time-resolved reflection spectroscopy has revealed the ultrafast timescale of the crystallization pathway^3^,^4^,^5^. Because the spatial resolution of optical techniques is on the order of micrometre, time-resolved X-ray diffraction can be invoked to elucidate the non-thermal structural phase transitions, such as melting^6^,^7^ and solid–solid transformation^8^. The kinetics of growth (crystallization) measured by diffraction can be on the minute timescale^9^,^10^.

The process of liquid-to-crystal transformation involves, according to the phase diagram of the material (see below), several elementary steps, including the critical nucleation dynamics, and as such then occurs on different timescales. A fundamental issue here is whether crystal formation involves a free-energy surface of one barrier or does it manifest a two-step process with an intermediate structural state that is often poorly ordered, amorphous or even liquid^11^,^12^. From an experimental point of view, the dynamics for these processes would be of special relevance to many applications, especially if the material thickness is on the nanoscale.

We chose titanium dioxide (TiO_2_) as a prototype material for a number of reasons. Besides the fundamental issues raised above, it is relevant to energy and environmental applications^13^,^14^,^15^, and also in solar-energy conversion and photocatalysis^16^,^17^. Much effort has been devoted to studies of the physical and chemical properties of TiO_2_ that depend on its dimension, morphology and crystalline polymorphism: anatase and rutile phases^18^,^19^,^20^. The study of its nanoscale domains and their transformation from the melt to the solid phase could exhibit properties that are different from that of the bulk materials.

In this contribution, the *in situ* phase transition of TiO_2_ in a nanometre-thick film is observed, from the amorphous liquid phase to the crystalline phase, with the spatiotemporal resolutions of four-dimensional (4D) electron microscopy^21^. The scheme of single-pulse microscopy is depicted in Fig. 1. A single optical ultraviolet pulse was used to feed energy to the TiO_2_ thin film, prepared by magnetron sputtering, while each probe imaging pulse of an electron bunch recorded the diffraction of the transients involved at a well-defined time delay. The temporal behaviour of the degree of crystallinity exhibits unique ‘two-step dynamics' with a plateau that extends over a microsecond, as shown in Fig. 2. Based on the observed kinetic behaviour, together with the theoretical extension of the Johnson–Mehl–Avrami–Kolmogorov (JMAK) model, we are able to identify the nucleation regime, which is defined by the latent heat of crystallites, and provide the timescale for reaching this regime, and that of crystal formation.

## Results

### Spatiotemporal visualization

**Figure 1:**
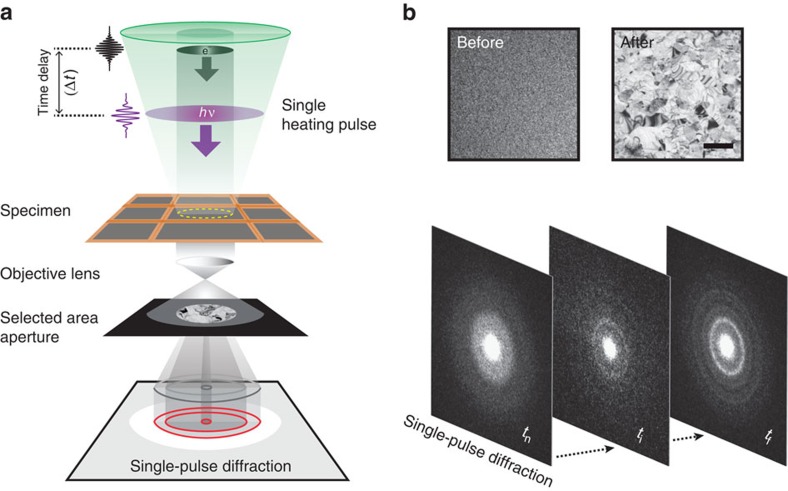
4D single-pulse microscopy. (**a**) The delayed e^−^ packet and the heating pulse enable the degree of crystallization to be measured by probing the increased intensity of diffraction at a given time delay. (**b**) Morphological (static) and structural (dynamic) transformation before and after the heating pulse is focused onto the amorphous TiO_2_ nanofilm. A series of 2D diffraction patterns for negative, intermediate and a final time frame reflects the degree of transformation; scale bar, 1 μm.

The scheme of single-pulse microscopy is depicted with the image changes of TiO_2_ before and after single-pulsed laser irradiation and time-resolved diffraction patterns at the given time delays in Fig. 1. In the bright-field images before the laser excitation, the diffraction is diffused with characteristics of the amorphous phase. Upon the single-pulsed irradiation (∼10 μJ per pulse; 120 mJ cm^-2^ at 355 nm), a marked change is clear in the bright-field images, which now displays grain formation with well-defined diffraction rings. Such behaviour elucidates the phase transformation: following the transition of the initial amorphous material into the liquid by the single-pulsed excitation, crystallites are formed at longer times.

**Figure 2:**
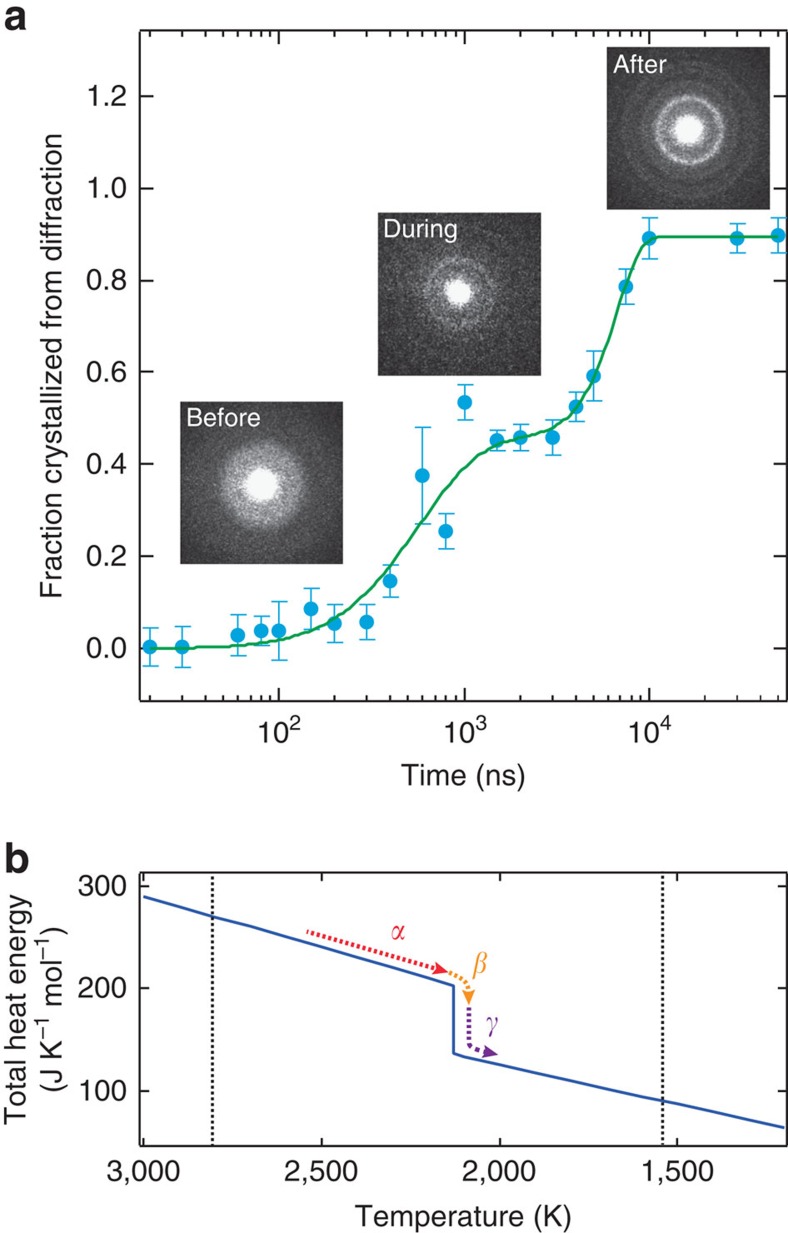
Kinetic profile of liquid-to-crystal phase transition and phase diagram of TiO_2_. (**a**) Time-dependent growth of the fraction crystallized obtained from the increased diffraction intensities. Single-pulse diffraction images are chosen and shown to be representative of time points before, during and after the processes are completed. The fraction versus time in log scale gives the kinetic profile of the structural transformation of TiO_2_ nanofilm. Circles are obtained from the average of individual measurements and error bars indicate the s.d. (**b**) Phase diagram: solid and liquid TiO_2_ against temperature based on thermal experiments. The distinct processes are labelled as *α*, *β* and *γ* to make the correspondence with our kinetic mapping. Note that the *x* axis for temperature decreases from left to right, and that the temperature range of our experiment is indicated by the dotted lines. The solid curve is the theoretically obtained behaviour (see text).

**Figure 3:**
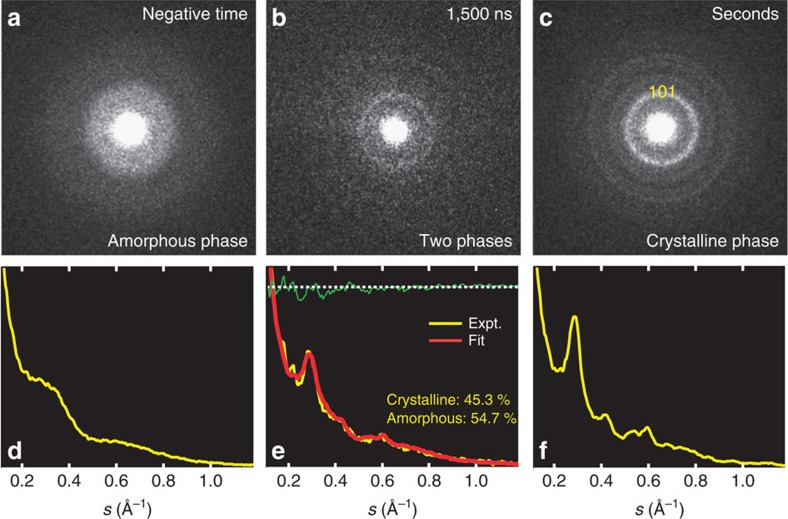
Transient diffraction profiles representing each phase. (**a–f**) Diffraction patterns (**a–c**) and their radially averaged profiles showing three phases at negative, intermediate and final times, which are obtained from the same area of irradiation (**d**–**f**). For a typical transient-frame diffraction (**b**,**e**), a linear combination of both diffractions at negative time and at seconds was used to represent the proportion of each contribution, as indicated in yellow. Our theoretical assignment and fit of the diffraction (101) is entirely consistent with TiO_2_ structure: 1/*s*=3.517 Å. Expt., experimental.

**Figure 4:**
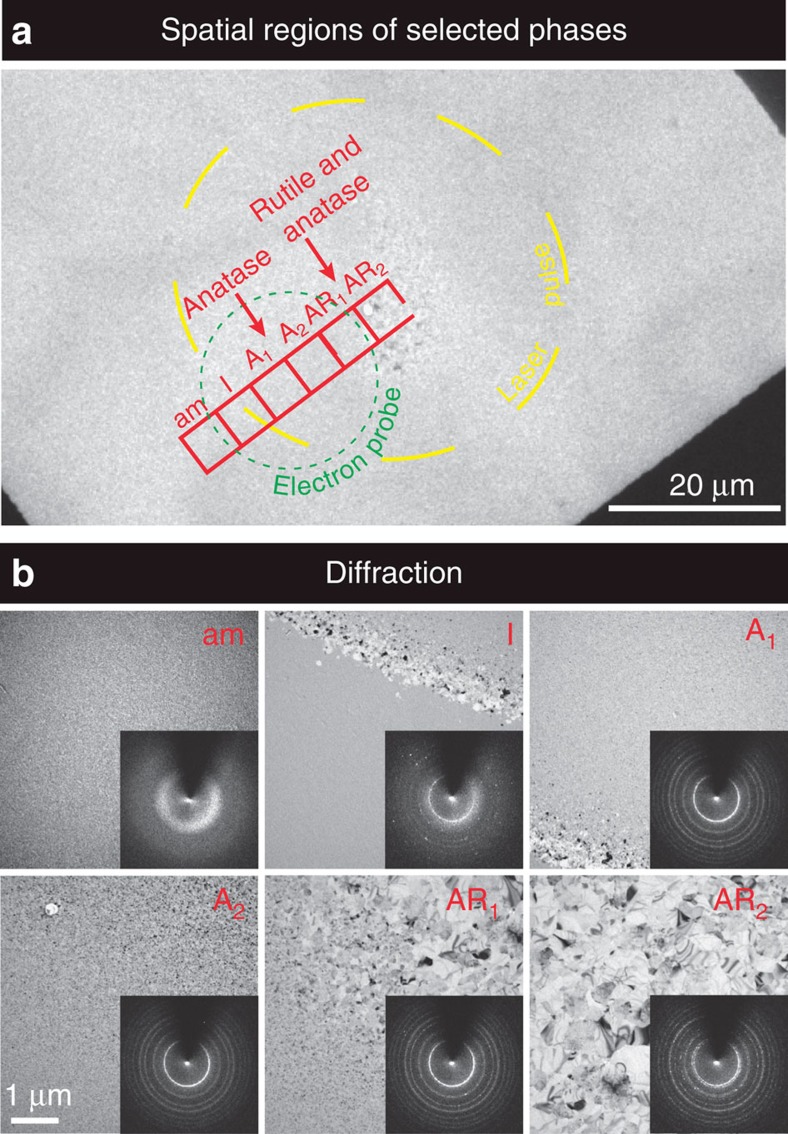
Images and diffractions for selected regions. (**a**) Bright-field images at low magnification and (**b**) those at higher magnification together with their corresponding diffraction patterns (inset) showing the spatial variation of the morphology of TiO_2_ after single-pulse laser irradiation. Yellow dashed line indicates the burn mark and red squares are chosen from the centre to the outer region to separate the phases spatially. Squares are divided to indicate each phase: the anatase–rutile mixture (AR_1,2_), the two serial anatase (A_1_–A_2_), the interface (I) and the amorphous (am). The probe area for our measurements is marked as a green dotted circle and also indicated in green arrow in calculated temperature profile (Supplementary Fig. 1).

In order to follow the entire process, the time delay is stepped and the crystallinity of the mass as a whole is measured from the diffraction, as shown in Fig. 2. Each transient diffraction frame at a given time delay (Fig. 3 and Supplementary Fig. 2) was obtained by translating the specimen so that each fresh specimen area is examined by the same excitation laser and the probe electron pulse, as shown in Fig. 1a. Diffraction data of at least three independent experiments were obtained and averaged to represent the change. The probe beam area was typically 22.7±1.5 μm in diameter and its centre was 15±3.0 μm off from the centre of the pump beam as shown by the green and the yellow circle, respectively, in Fig. 4a. This was done purposely to spatially separate the rutile from anatase phases of crystallization (Fig. 4). It is evident that diffraction rings obtained at very long times (s), that is, after relaxation, only represent the contribution of the anatase phase. At the shorter times reported here, the normalized diffraction to the total electron count provides the time-dependent ratio of the amorphous-to-anatase phase that was obtained. For example, transient diffraction profile at 1,500 ns (noted by yellow in Fig. 3e) is well fitted using a linear combination of two profiles: one at negative time (54.7±4.3%) and another at seconds (45.3±4.3%; refs 22, 23). To construct the kinetics of interconversion between two distinct phases, the same procedure was carried out for all acquired diffraction data (Supplementary Fig. 3). We repeated the measurements many times at each delay time, and obtained the results in Fig. 2.

**Figure 5:**
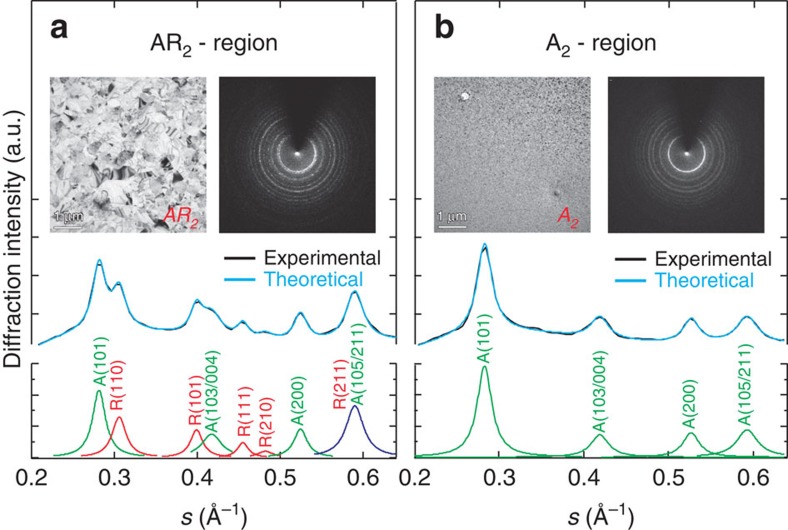
Static diffraction pattern analysis. Morphological (image) and structural (diffraction) differences in two representative areas: (**a**) AR_2_ and (**b**) A_2_ with their radial diffraction intensity distribution. Radial diffraction profiles are fit with a sum of Gaussian peaks and each peak is assigned according to known Miller index values of TiO_2_. Anatase (green) and rutile (red) peaks coexist in **a**, and only anatase peaks are found in **b**. Unresolved peak in violet is a mixture of the two phases.

The separation of the rutile and anatase phases is evident in the experiments done selectively on the different spatial regions noted by the red boxes in Fig. 4a. The diffraction given in Fig. 5 is for two of such regions, namely AR_2_ (anatase and rutile at the centre) and A_2_ (only anatase), and clearly fits the theoretical diffraction indexed according the data obtained from X-ray analysis^24^. Such separation of phases enables us to study the dynamics of crystallization for the transformation into the specific phase, anatase.

Finally, we obtained the rates of the processes involved at each time delay. In Fig. 2a, one can see two robust plateaus for the transformation. The first plateau bridges two distinct rate processes, determined by the specific time delays shown in the Supplementary Fig. 4. The theoretical curve (green) was obtained from the analytical model, to be discussed below, and the fit is satisfactory over the entire time range of 50 μs. Quantitatively, from the data in Fig. 2, obtained were three time constants of 294 ns (*α*^−1^), 271 ns (*β*^−1^) and 6.72 μs (*γ*^−1^), together with the degree of crystallization (*η*), which was found to vary from 0 to 0.9; at the first plateau, *η* is 0.45. The plateau extends over the 1-μs range, and at longer times, structural crystallinity reaches its maximum value when *η*=0.9 at the second plateau. The slope for reaching the first plateau is less steep than that of the second plateau. The origin of this disparity will be discussed below within the framework of the theory presented.

### The T-jump and area-selected diffraction

In this study, the phase transition is induced by laser heating at 3.5 eV (*λ*=355 nm), which exceeds the bandgap energy of TiO_2_ by 0.3 eV (ref. 16). The electronic excitation leads to the population of electrons in the conduction band of the semiconductor. With sufficiently high-pump fluence, the temperature of illuminated areas rise above the threshold temperature for phase change, following electron–electron scattering and electron–phonon relaxation on the timescale of up to a few picoseconds^25^. Accordingly, on the timescale of nanosecond, the energy needed to crystallize TiO_2_ is provided as a temperature jump, Δ*T*, or heat energy transfer from the substrate into the material.

The temperature rise can be estimated from knowledge of the fluence and the known thermodynamic and optical properties of the material/substrate. Because TiO_2_ was deposited directly on a SiO substrate, it is necessary to consider both materials to calculate the actual temperature increase, Δ*T*. We found that the temperature of TiO_2_ reaches 2,564±255 K (Supplementary Note 2). At the probed area for time-framed diffraction patterns (Fig. 4), the centre of which is 15 μm off from the centre of the pump beam, which has a 22.7±1.5 μm diameter, the maximum averaged temperature was estimated to be 2,166±193 K, which is consistent with the melting point *T*_m_ of TiO_2_ (2,130 K; ref. 26).

Melting of materials' films occurs on the picosecond timescale^27^, and, consequently, the quenching of the melt results in the recrystallization during cooling down. In case of semiconductors, such as silicon, the nanosecond annealing is achieved via the liquid phase of silicon and its subsequent epitaxial regrowth^22^. Noticeably, grain size enlargement is apparent at the centre (Fig. 4b), and this is a general phenomenon often found in melt-mediated recrystallization processes^28^.

Amorphous TiO_2_ films are known to crystallize into the anatase phase at 600–800 K, under low thermal activation energy, whereas those annealed at 1,400 K result in a rutile structure^29^. In thermal heating, the grains begin to grow until they coarsen and fuse to form large crystallites. However, the annealing picture is different when the heating rate becomes 10^8^ orders of magnitude faster than that of conventional heating. Here the pulsed heating beam with a Gaussian spatial profile results in the spatial distribution of crystallinity in the specimen. The rutile appears in the centre because the anatase anneals in the hotter region. At the core (AR_2_) area in Fig. 4b, the portion of the rutile phase is present but is negligible in AR_1_. Differently shaped crystallites are formed by the randomly distributed orientations of the crystal nuclei in AR_2_ and AR_1_ region. There is no direct measurement of transformation from the amorphous-to-rutile phase by thermal annealing, but only via anatase. Our laser annealing provides the possibility to synthesize nano-composite films containing both phases by a single pulse for a desired space location and with well-defined time.

## Discussion

The crystallization process from the melt may involve a one-step barrier crossing or a two-step nucleation pathway as discussed in refs 11, 12. Our observation of two successive ‘S-shaped' curves for the degree of crystallinity as a function of time (Fig. 2a) indicate the presence of an intermediate structure on the free-energy surface—it is the nucleation step observed in real time. For decades, the JMAK model has been used to describe and analyse ‘crystal growth' time profile, which is characterized by a stretched-exponential function. Here, because of the observed two steps, we propose a model that accounts for the presence of a metastable state in the nucleation process; when reaching this state, crystallization begins from an inhomogeneous ensemble as evident by the fast temporal ‘stretched-exponential' behaviour.

For a general single-exponential process with a lifetime *τ*_*c*_, and an initial value of zero, having an asymptotic plateau value of *c*, we can express the increase in crystallinity using the following simple equation:


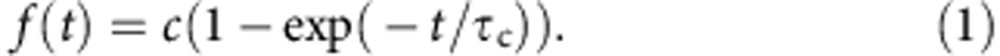


However, where a distribution of lifetimes exists, one needs to sum up all the system's constituents as shown in equation (2). Then, we can substitute the summation by an integral over the whole system with a weighting function *J*(*ω*) over *ω*, which is the inverse of the lifetime as shown in equation (3). By defining *ω*_*k*_=1/*τ*_*c,k*_ and assuming *J*(*ω*) of Havriliak and Negami^30^ we obtain


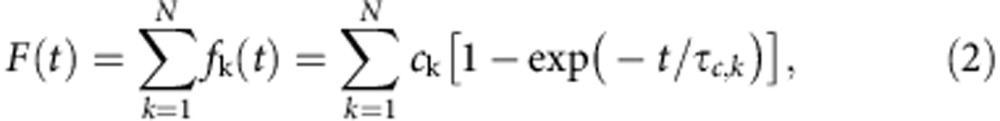



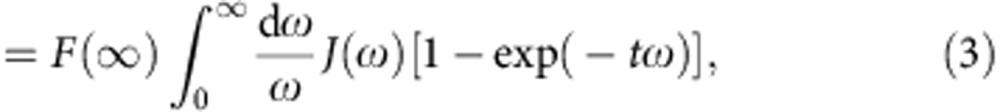



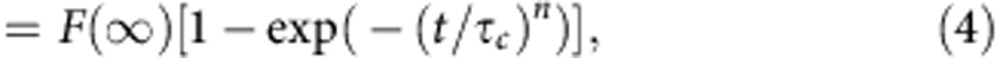


where 
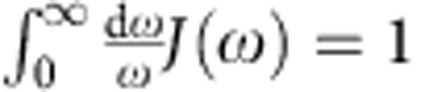
 for the normalized distribution density function. Equation (4) shows the stretched-exponential behaviour of the well-known JMAK equation^31^ for crystal growth. That model is often used to obtain the kinetic parameters related to phase transitions using *F*(*t*)=1−exp(−(*kt*)^*n*^), where *F* is the crystallization fraction, *k* is a rate constant, *t* is the time required to reach the crystallization fraction and *n* is the Avrami exponent that reflects how the transformation propagates through materials^31^.

Based on the known experimental phase diagram (Fig. 2b; ref. 26) for the solid and liquid phases of TiO_2_, we can characterize four distinct states within the temperature range of our study: |1> the liquid state at a temperature higher than *T*_m_; |2> the liquid state at *T*_m_, but with a latent heat *H*_L_ just before nucleation; |3> the nucleation state at *T*_m_ with the fraction crystallized *η*; that is, a mixture of crystalline and liquid TiO_2_, with partially dissipated latent heat; and |4> the fully grown solid state with crystalline grains when the latent heat dissipates to the surroundings.

Let us denote three heat dissipation rates: *α*, *β* and *γ* for |1> to |2>, |2> to |3> and |3> to |4>, respectively. They are displayed in the phase diagram as arrows in Fig. 2b. The corresponding kinetic equations can be solved analytically by the Laplace transform method in a closed form (Supplementary Note 1). The time dependence of the fraction crystallized (Supplementary equation 5) can be simplified to yield:


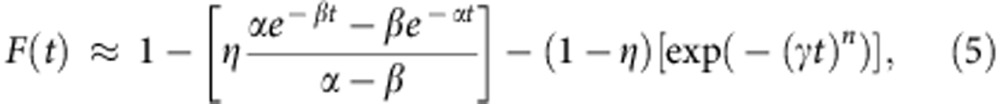


with *α*, *β* and *γ* (rate constants), *η* (degree of crystallization) and *n* (Avrami exponent). Our results exhibit two plateaus, and the existence of the intermediate plateau is necessary for the nucleation stage when the latent heat and entropy are decreasing.

Up to the first plateau, two rates (*α* and *β*) are controlling the formation of the intermediate step with *F*(*t*) describing the bi-exponential behaviour: *α*^−1^ (294 ns) is the time for the process involving the hot-liquid TiO_2_ reaching isothermal temperature (2,130 K; Fig. 2b), whereas the faster *β*^−1^ (271 ns) defines the nucleation when the latent heat is lowered by action of the surroundings. On the other hand, the relatively long-time behaviour (6.72 μs for *γ*^−1^) corresponds to the time for crystal formation obtained from the behaviour observed from the first-to-second plateau. Our results for this region demonstrates a stretched-exponential rise having *n*=3.5. In the JMAK model, it is generally known that *n*=1, but when *n*>1, both the nucleation and the growth step are involved in the transformation^32^,^33^. For TiO_2_, using thermal heating, *n* is in the range of 2–3 (ref. 34). We note that the studies made here are selective in their spatial and temporal resolution of crystallization, and offer, through structural changes (diffraction), comparisons with optical reflectivity (dielectric) methods^5^,^35^,^36^.

Even though the existence of a two-step process is consistent with the unique observations made here, the results have some interesting implications. For protein systems, the two-step pathway is inherent to the system, but a direct pathway is unlikely to be observed because order cannot arise before the densification occurs^37^. In case of TiO_2_ with a small-enough nanoscale particle size, the two-step pathway has been identified in the thermodynamic equilibrium state^38^. However, with ultrafast heating, the picture may be different; rapid heating could lead to a kinetically trapped amorphous state. In the thermodynamic state, experimental studies, using levitator traps, together with theoretical advances, have elucidated the presence of a two-step nucleation; for reviews, see refs 39, 40, 41. The above discussion thus indicates that future experiments on different timescales, and using *in situ* liquid cells, will further elucidate the mechanism of crystallization in different systems.

The reported spatiotemporally resolved observations of the steps involved in an irreversible crystallization processes, together with the theoretical model that accounts for the two distinct elementary regions of the phase diagram, elucidate the nature of crystal nucleation and growth as they occur in both space and time. Here, with the time resolution of diffraction (structure), we obtain the rate for the liquid to reach the isothermal (constant temperature) region, that of nucleation when the latent heat is lowered by the surroundings and nucleation ensues, and finally crystal formation. This approach has the potential for many applications in materials science and possibly for biological specimens. Direct structural imaging provides the key parameters for the transient behaviour of crystallinity at the nanoscale.

## Methods

### Preparation of TiO_2_ thin films

Amorphous TiO_2_ films were uniformly obtained by Radio Frequency (RF) magnetron sputtering directly onto 200-mesh copper TEM grids with silicon monoxide as a substrate^42^. Several TEM grids were placed in the home-made sample holder (steel) and carried into the sputtering chamber (AJA international, Scituate, MA). A TiO_2_ sputtering disk (ACI alloys, Inc., San Jose, CA) with a purity of 99.999% was used as a target under a low pressure (1.5 mTorr) at room temperature. Duration for sputtering was controlled by changing deposition time to optimize the condition for subsequent laser processing. The thickness (88±5.0 nm) of TiO_2_ deposited on a TEM grid was measured by electron energy loss spectroscopy. Both the electron energy loss spectra and the diffraction patterns of the prepared sample clearly verified the deposition of amorphous TiO_2_ on the substrate with high purity.

The crystallization of TiO_2_ was initiated by 355-nm ns laser pulses that were focused onto the specimen in our microscope. Consequent structural evolution of amorphous TiO_2_ before and after the single-pulse irradiation is depicted in Fig. 1b. Images of higher magnification reveal the different morphology having tens of nanometre-sized particles after the heating pulse from that of the as-deposited film. The diameter of the burn mark was found to depend on the input energy of the excitation pulse, which was optically monitored and estimated to have the full-width at half-maximum of 55±3.0 μm, assuming its 2D profile to follow a Gaussian distribution in intensity. The average aspect ratio of the elliptical patterns was obtained to be ∼1.16:1, and their mean diameter was estimated to be 48 μm from visual inspection of a random selection of irradiated specimens: it varied from 36 to 59 μm, depending on the energy of the excitation pulse.

### Single-pulse microscopy

The TiO_2_ films were imaged in our 200-kV 4D electron microscope using an optical pump beam and the photoelectron probe beam^43^. Time-resolved experiments were carried out in a single-shot mode of operation^22^. The two ns lasers for the pump and probe beams for each shot were Q-switched, diode-pumped, all-solid-state Nd:YAG lasers, which operate at the wavelengths of 355 and 266 nm. The 355-nm laser (pulse length ∼10 ns) was used to excite the specimen, whereas the 266-nm laser was used to generate the pulsed photoelectrons, which were accelerated to 200 kV.

Time zero was defined by examining the morphological response in the dark-field images of the TiO_2_ specimen pumped by the 355-nm laser pulses. The temporal delay was controlled from 300 ns before the time zero to seconds after the time zero, if necessary, by electronic triggering to cover the full dynamic range of the phase transition. In the single-pulse mode of operation, an entire diffraction image is formed with only one pulse of many electrons. All single-pulse diffraction data were obtained by adjusting the probe area to be 22.7±1.5 μm in diameter on the specimen. Each single pulse for diffraction measurements has ∼10^5^ electrons. Details of the procedure are described in previous studies from this group^22^,^44^.

There is no crystallization of the amorphous TiO_2_ below the threshold pump energy. The pump-beam diameter is also optically controlled to consider this threshold. Both the areas of pump and probe beams are adjusted to be micron sized. When performing single-shot experiments, we selectively chose the probe beam area 15±3.0 μm away from the centre of pump beam to minimize the contribution of the rutile phase in the measured diffraction patterns. Thus, we only probe crystallization from the amorphous to the anatase phase. The variation in beam position measured during the course of a day's experiment has a s.d. of 2.97±0.07 μm. To reduce artefact arising from the possible ellipticity of recorded diffraction patterns, diffraction-ring distortion correction was performed for all the raw diffraction data^45^.

## Additional information

**How to cite this article:** Yoo, B.-K. *et al*. Observing in space and time the ephemeral nucleation of liquid-to-crystal phase transitions. *Nat. Commun*. 6:8639 doi: 10.1038/ncomms9639 (2015).

## Supplementary Material

Supplementary Information Supplementary Figures 1-4, Supplementary Notes 1-2 and Supplementary References
