# Supplementary material for: Observing in space and time the ephemeral nucleation of liquid-to-crystal phase transitions
Source: Nat Commun. 2015 Oct 19;6:8639. doi: 10.1038/ncomms9639 (PMC4667692; doi:10.1038/ncomms9639)
Supplement: Supplementary Information — Supplementary Figures 1-4, Supplementary Notes 1-2 and Supplementary References [file ncomms9639-s1.pdf]

Supplementary Figures

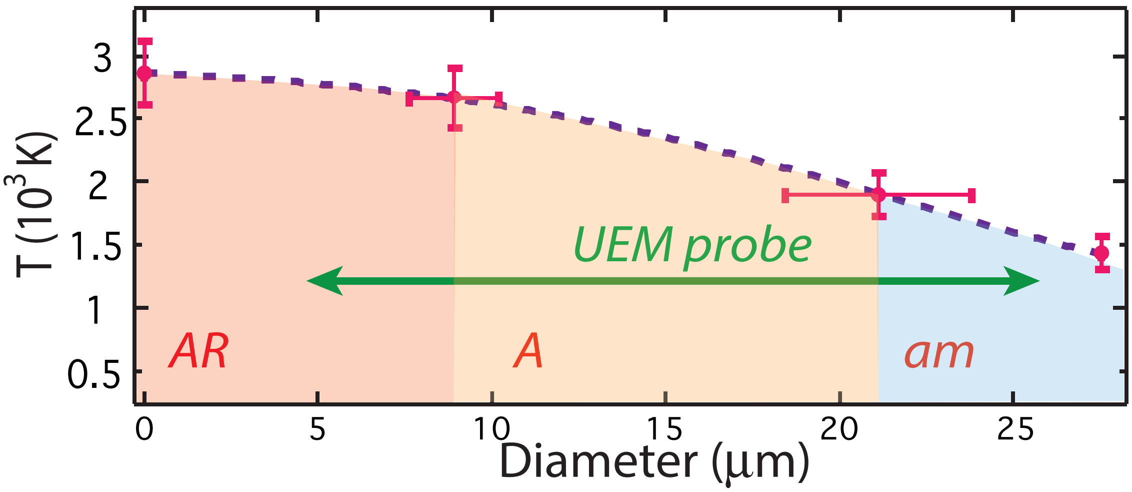

Supplementary Figure 1. Calculated Gaussian temperature distribution vs a radial distance from the maximum is presented to estimate the radius of each phase in the burn mark. Selected area diffraction investigation determines the phase distribution vs diameter within the region of interest.

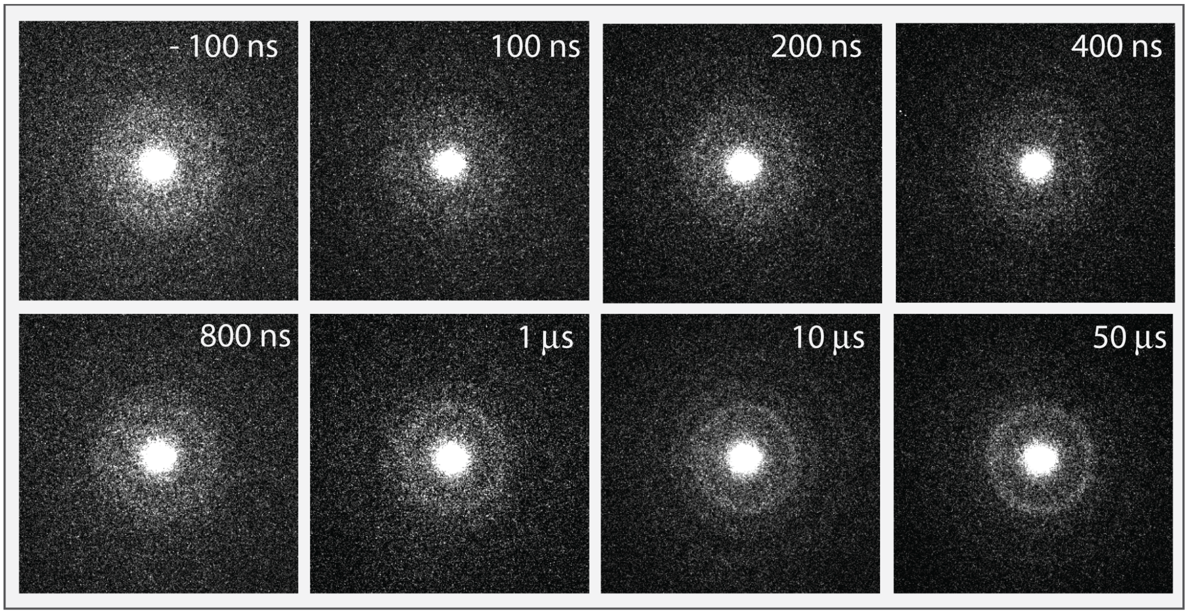

Supplementary Figure 2. Snapshots of the transformation at representative time delays. Single-pulse diffraction images that have an exposure time of 10 ns at different time delays are shown to elucidate the enhancement of scattering with time.

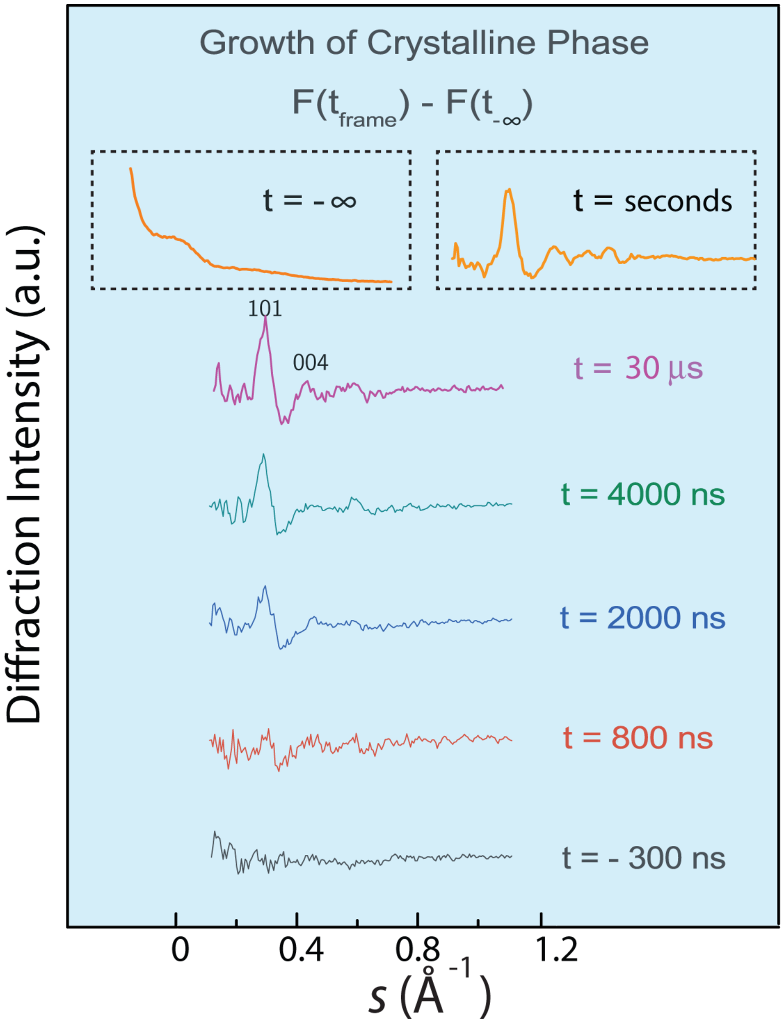

Supplementary Figure 3. Real-time evolution of the crystalline phase obtained by using single-pulse imaging. Transient-frame diffraction profiles are displayed for each representative delay time. The contribution (box, left) of amorphous diffraction taken before heating ($t = -\infty$) was subtracted. Diffraction grows in amplitude for all frames with time, while the diffraction at seconds (box, right; chosen for 2000 ns) is the same for all time delays.

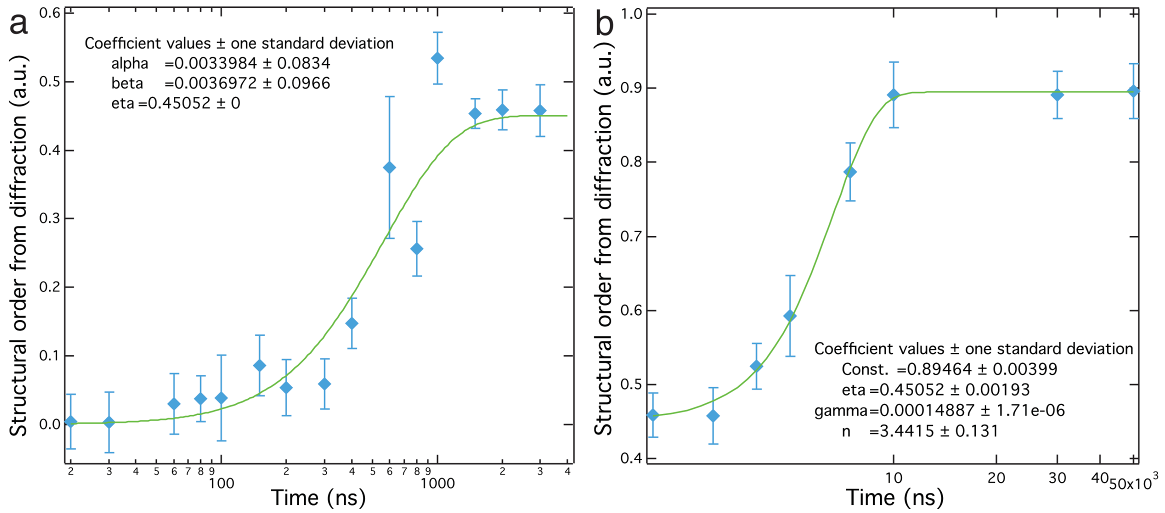

Supplementary Figure 4. Analytical fitting of separate time windows **a)**: up to the first plateau, **b)**: first-to-second plateau) gives each value for α, β, γ (rate constants), and η (degree of crystallization), together with n (Avrami exponent) and constant (C). Experimental data are fitted with $F(t) = \eta\left[1 - \frac{\alpha e^{-\beta t} - \beta e^{-\alpha t}}{\alpha - \beta}\right]$ for **a** and $F(t) = C - (C - \eta)[\exp(-(\gamma t)^n)]$ for **b**, respectively.

Supplementary Notes

Supplementary Note 1. Analytical modeling: rate equations and Laplace transform method in a closed form

Each fraction for four distinct states (|1> to |4>) is given as f_i, together with three rate constants ($\alpha, \beta,$ and γ) to represent the evolution kinetics of each state. The heat dissipation rates for each transformation can be described using the following coupled rate equations for each fraction (f_i) as

$$\frac{d}{dt}f_1 = -\alpha f_1, \qquad \text{(Supplementary Equation 1)}$$

$$\frac{d}{dt}f_2 = \alpha f_1 - \beta f_2, \qquad \text{(Supplementary Equation 2)}$$

$$\frac{d}{dt}f_3 = \beta f_2 - \gamma f_3, \qquad \text{(Supplementary Equation 3)}$$

$$\frac{d}{dt}f_4 = \gamma f_3, \qquad \text{(Supplementary Equation 4)}$$

with the initial condition for population at time zero, $f_1(0) = 1, f_2(0) = f_3(0) = f_4(0) = 0$. Taking η to express crystallinity with values between 0 and 1, one can relate the overall fraction of transformation $F(t) = \eta f_3(t) + f_4(t)$ by solving the above coupled rate equations. The three rate constants ($\alpha, \beta,$ and γ) and the crystallinity (η) can be obtained using the following formula obtained by the Laplace transform method:

$$F(t) = \frac{\beta(\alpha\eta-\gamma)e^{-\alpha t}}{(\alpha-\beta)(\alpha-\gamma)} + \frac{\alpha(\beta\eta-\gamma)e^{-\beta t}}{(\beta-\alpha)(\beta-\gamma)} + \frac{\alpha\beta(\eta-1)e^{-\gamma t}}{(\gamma-\alpha)(\gamma-\beta)} + 1, \qquad \text{(Supplementary Equation 5)}$$

which represents the time dependence of the overall crystallization ordering parameter. The above equation (Supplementary Equation 5) can be simplified to give,

$$F(t) \approx \eta\left[e^{-\gamma t} - \frac{\alpha e^{-\beta t} - \beta e^{-\alpha t}}{\alpha-\beta}\right] + [1 - e^{-\gamma t}], \qquad \text{(Supplementary Equation 6)}$$

when γ is much smaller than α and β. During the initial stage when $t \ll 1/\gamma$, this equation is further simplified to

$$F(t) \approx \eta \left[1 - \frac{\alpha e^{-\beta t} - \beta e^{-\alpha t}}{\alpha - \beta}\right], \qquad \text{(Supplementary Equation 7)}$$

which covers up to the first plateau, having partial crystallization order (η). At a later time with $t \gg 1/\alpha$ and $1/\beta$, equation (Supplementary Equation 6) can be approximated as

$$F(t) \approx 1 - (1 - \eta)e^{-\gamma t}. \qquad \text{(Supplementary Equation 8)}$$

For a heterogeneous system with a distribution of the growth rate γ, one can modify equation (Supplementary Equation 8) by a stretched exponential as

$$F(t) \approx 1 - (1 - \eta)[exp(-(\gamma t)^n)], \quad \text{(Supplementary Equation 9)}$$

and likewise, equation (Supplementary Equation 6) can be generalized to

$$F(t) \approx 1 - \left[\eta \frac{\alpha e^{-\beta t} - \beta e^{-\alpha t}}{\alpha - \beta}\right] - (1 - \eta)[exp(-(\gamma t)^n)]. \quad \text{(Supplementary Equation 10)}$$

Using equations (Supplementary Equation 7-8), the crystallization ordering parameters at separate time windows were fit successfully as shown in Supplementary Fig. 4. For the rise covering the first-to-second plateau, the fit was performed using stretched exponential function that arises naturally in many physical processes including crystal growth as well as dielectric relaxation in heterogeneous systems.[1] On the other hand, for the rise of the first plateau we could fit the experimental data with the bi-exponential kinetics of α and β. Once a full crystal is formed any heat dissipation below the melting point is not able to change the ordering parameter, and further cooling is related to the length of the second plateau and is not relevant to the experiments.

Supplementary Note 2. Temperature of the liquid, solid, and the substrate

To quantify the experimental observation of laser-induced melting and recrystallization, it is necessary to estimate the temperature changes in the TiO_2 thin film sample as well as the substrate. The light at 355 nm is partially reflected by the surface of amorphous TiO_2; 27 % of the light is reflected.[2-4] Because the measured absorption coefficient for amorphous TiO_2 at 355 nm is $\alpha_T \sim 3.5 \times 10^4$ cm^{-1},[5] the laser penetration depth is found to be ~286 nm, which exceeds the thickness of the layer (88 nm), and thus in what follows we shall consider both the nano-film and the substrate.

For a light pulse of peak fluence F_0 and $R_\mathrm{T} = (\frac{1-n_\mathrm{T}}{1+n_\mathrm{T}})^2$ with the refractive index n_T, in a layer of thickness L_T, the heat absorption for the amorphous TiO_2, ΔH_T, is given by

$$\Delta H_\mathrm{T} = (1 - R_\mathrm{T})F_0 \frac{1-exp(-\alpha_\mathrm{T} L_\mathrm{T})}{L_\mathrm{T}} = \left(T_{TiO_2} - 298\right) C_{p,T_\mathrm{solid}}. \quad \text{(Supplementary Equation 11)}$$

Subscripts (T, S) indicate TiO_2 and SiO, respectively. Here, C_{p,T_solid} is the heat capacity of $2.9 \times 10^6\ \mathrm{J/m^3 \cdot K}$ for solid TiO_2,[6] L_T is the thickness of 88 ± 5.0 nm for the deposited TiO_2. $T_{\mathrm{TiO_2}}$ is found to have 2125 ± 121 K.

Because the penetration depth is larger than the thickness of TiO_2 and SiO (23 ± 2.5 nm), both contribute to the temperature increase. We use the approach described in Ref. 7 to calculate the temperature.[7] The heat absorption (ΔH_S) on the substrate up to the time (τ_S) is given by

$$\Delta H_\mathrm{S} = (1 - R_\mathrm{S})F_0 exp(-\alpha_\mathrm{T} L_\mathrm{T}) \frac{1-exp(-\alpha_\mathrm{S} L_\mathrm{S})}{L_\mathrm{S}} \times \int_{-\infty}^{\tau_\mathrm{S}} f(t)dt$$

$$= (1 - R_\mathrm{S})F_0 exp(-\alpha_\mathrm{T} L_\mathrm{T}) \frac{1-exp(-\alpha_\mathrm{S} L_\mathrm{S})}{L_\mathrm{S}} \times \frac{1 + erf(\frac{\tau_\mathrm{S}}{\sqrt{2}\sigma})}{2}. \quad \text{(Supplementary Equation 12)}$$

The first portion of the heat absorbed is for the temperature rise in the thin layer of the solid phase from room temperature of 298 K to the phase transition temperature T_c of 2550 K between crystalline and liquid of SiO.[8] Here, R_S is $(\frac{n_\mathrm{T}-n_\mathrm{S}}{n_\mathrm{T}+n_\mathrm{S}})^2$ considering the bilayer of TiO_2 and SiO

(refractive index n_S). The latent heat for the subsequent phase transition to the liquid phase is made equal to ΔH_S by the following expression:

$$(1-R_S)F_0 exp(-\alpha_T L_T)\frac{1-exp(-\alpha_S L_S)}{L_S}\times\frac{1+erf(\frac{\tau_S}{\sqrt{2}\sigma})}{2} = \Delta H_{L,S} + (T_c - 298)C_{p,S_{solid}},$$

(Supplementary Equation 13)

where α_S is the absorption coefficient ($9.05\times10^4 cm^{-1}$ at 355 nm),[9] $\Delta H_{L,S}$ is the latent heat of 2.40×10^9 J/m^3,[8] $C_{p,S_{solid}}$ is the heat capacity of 1.44×10^6 J/m$^3\cdot$K for solid SiO.[10] L_S is the thickness of 23 ± 2.5 nm and n_S is the refractive index of 2.14 for SiO. For the remaining portion of the Gaussian pulse, from time τ_S and afterward, the heat ($\Delta H_{S,liquid}$) absorbed by the liquid phase of SiO is given by

$$\Delta H_{S,liquid} = (1-R_S)F_0 exp(-\alpha_T L_T)\frac{1-exp(-\alpha_S L_S)}{L_S}\times\int_{\tau_S}^{\infty} f(t)dt$$

$$= (1-R_S)F_0 exp(-\alpha_T L_T)\frac{1-exp(-\alpha_S L_S)}{L_S}\times\frac{1-erf(\frac{\tau_l}{\sqrt{2}\sigma})}{2} = (T_{SiO} - T_c)C_{p,S_{liquid}},$$

(Supplementary Equation 14)

where the refractive index and absorption coefficient are used the same as those of solid SiO, $C_{p,S_{liquid}}$ is the heat capacity of 2.77×10^9 J/m^3 for liquid SiO.[10,11] After the value of τ_s is calculated from equation (Supplementary Equation 13), the only unknown parameter is that of the final temperature reached in the layer caused by laser heating in equation (Supplementary Equation 14) is given T_{SiO} = 5546 ± 616 K. The final temperature at equilibrium (T_e) from two temperatures is found to be 2862 ± 255 K using following equation:

$$(T_{SiO} - T_e)\times C_{p,S_{liquid}} = C_{p,T_{solid}}(T_m - T_{TiO_2}) + \Delta H_{L,T} + (T_e - T_m)C_{p,T_{liquid}},$$

(Supplementary Equation 15)

where $C_{p,T_{liquid}}$ is 5.3×10^6 J/m$^3 \cdot$ K for liquid TiO_2 and the latent heat $\Delta H_{L,T}$ is 3.54×10^9 J/m^3 of TiO_2[11] T_m is the melting point of TiO_2 (2130 K).[12]

Supplementary References

1. Phillips JC. Stretched exponential relaxation in molecular and electronic glasses. *Rep. Prog. Phys.* **59**, 1133-1207 (1996).

2. Černý R, Přikryl P. Chapter 3 Modeling Laser-Induced Phase-Change Processes: Theory and Computation. In: *Semiconductors and Semimetals* **75**, 43-78 (ed. Prof, H.N.). Elsevier (2003).

3. Zhao Z, Tay BK, Yu G. Room-temperature deposition of amorphous titanium dioxide thin film with high refractive index by a filtered cathodic vacuum arc technique. *Appl. Opt.* **43**, 1281-1285 (2004).

4. Palik ED. *Handbook of Optical Constants of Solids*. Academic Press, New York (1985).

5. Karunagaran B, Kumar RTR, Viswanathan C, Mangalaraj D, Narayandass SK, Rao GM. Optical constants of DC magnetron sputtered titanium dioxide thin films measured by spectroscopic ellipsometry. *Cryst. Res. Technol.* **38**, 773-778 (2003).

6. Shomate CH. Heat Capacities at Low Temperatures of Titanium Dioxide (Rutile and Anatase). *J. Am. Chem. Soc.* **69**, 218-219 (1947).

7. Liu HH, Kwon OH, Tang J, Zewail AH. 4D Imaging and Diffraction Dynamics of Single-Particle Phase Transition in Heterogeneous Ensembles. *Nano Lett.* **14**, 946-954 (2014).

8. Weast RC. Handbook of chemistry and physics, Cleveland. *CRC Press, Inc* **1975**, E58 (1974).

9. Hass G, Salzberg CD. Optical Properties of Silicon Monoxide in the Wavelength Region from 0.24 to 14.0 Microns. *J. Opt. Soc. Am.* **44**, 181-187 (1954).

10. Linstrom PJM, W. G. NIST Chemistry WebBook, NIST Standard Reference Database Number 69; NIST. (2010).

11. Chase MW. *NIST-JANAF themochemical tables, J. Phys. Chem. Ref. Data, Monograph 9*, 4th edn (1998).

12. Manion JA*, et al.* NIST chemical kinetics database, NIST standard reference database 17, version 7.0 (web version), release 1.4. 3, data version 2008.12, National Institute of Standards and Technology, Gaithersburg, Maryland, 20899-8320. *Web address: http://kinetics.nist.gov*, (2008).
